# Estimating the potential impact of a health tax on the demand for unhealthy food and beverages and on tax revenue in India

**DOI:** 10.1093/heapol/czad117

**Published:** 2023-12-15

**Authors:** Beena Varghese, Rajashree Panicker, Dripto Mukhopadhyay, Kathryn Backholer, Vani Sethi, Arjan de Wagt, Zivai Murira, Neena Bhatia, Monika Arora

**Affiliations:** Indian Institute of Public Health, Public Health Foundation of India, Rajendra Nagar Mandal, Kismatpur, Hyderabad, Telangana 500030, India; Total Alliance Health Partners International, Dubai Healthcare City, Dubai 66566, UAE; Ascension Centre for Research and Analytics (ACRA), 5/1, Sector 5, Rajendra Nagar, Sahibabad, Ghaziabad, UP 201005, India; A/P Deakin University, Geelong, Global Centre for Preventive Health and Nutrition, Institute for Health Transformation, Burwood, Victoria 3125, Australia; UNICEF Regional Office for South Asia, Kathmandu 5815, Nepal; UNICEF, New Delhi 110003, India; UNICEF Regional Office for South Asia, Kathmandu 5815, Nepal; Ministry of Health and Family Welfare, Government of India, New Delhi 110011, India; Public Health Foundation of India, Gurgaon, Haryana 122102, India

**Keywords:** Taxation, unhealthy foods, sugar, price elasticity, India, NCDs

## Abstract

Foods high in fat, sugar or salt are important contributors to the rising burden of non-communicable diseases globally and in India. Health taxes (HTs) have been used by over 70 countries as an effective tool for reducing consumption of sugar sweetened beverages (SSBs). However, the potential impacts of HTs on consumption and on revenues have not been estimated in India. This paper aims to estimate the potential impact of health taxes on the demand for sugar, SSBs and foods high in fat, sugar or salt (HFSS) in India while exploring its impact on tax revenues. PE of sugar was estimated using Private Final Consumption Expenditure and Consumer Price Index data while price elasticities for SSBs and HFSS were obtained from literature. The reduction in demand was estimated for an additional 10–30% HT added to the current goods and services tax, for varying levels of price elasticities. The results show that for manufacturers of sweets and confectionaries who buy sugar in bulk and assuming a higher price elasticity of −0.70, 20% additional HT (total tax 48%) would result in 13–18% decrease in the demand for sugar used for confectionaries and sweets. For SSBs, HT of 10–30% would result in 7–30% decline in the demand of SSBs. For HFSS food products, 10–30% HT would result in 5–24% decline in the demand for HFSS products. These additional taxes would increase tax revenues for the government by 12–200% across different scenarios. Taxing unhealthy foods is likely to reduce demand, while increasing government revenues for reinvestment back into public health programmes and policies that may reduce obesity and the incidence of non-communicable diseases in India.

Key messages

- Sugar sweetened beverages (SSBs) and foods high in fat, sugar or salt are important contributors to the rising burden of non-communicable diseases globally and in India.
- While over 70 countries are using ‘sin’ tax as an effective tool for reducing consumption of SSBs and unhealthy foods, the existing goods and service tax (GST) in India does not differentiate between healthy and unhealthy beverages.
- An additional ‘health’ tax component may be added to GST, which is adjusted annually to inflation and increase per capita income.
- Decline in consumption would not have a negative effect on tax revenues. Rather, tax revenues could potentially bring revenue for governments, which could be reinvested back into public health programmes and policies.

## Introduction

Overweight and obesity account for 4 million deaths annually with the global rates having tripled since 1975 (Shekar and Popkin, 2020). Almost three-fourths of these deaths are in low- and middle-income countries. In India, the recent national nutrition survey of the urban population found that more than half of the adults were overweight and obese (National Nutritional Monitoring Bureau, 2017). The global annual cost^1^ of overweight and obesity is projected to reach about US$7 trillion in the next 15 years (Shekar and Popkin, 2020; Abay *et al.*, 2022). Sugar sweetened beverages (SSBs) have emerged as an important risk factor, with a robust body of evidence linking SSBs to tooth decay, weight gain, childhood obesity, risk of type 2 diabetes mellitus, cardiovascular diseases, and certain cancers (World Bank, 2020; Malik and Hu, 2022). Dasgupta *et al*. reported highly significant association (dose–response) between per capita consumption of sugar, salt and fat in men and women, with the occurrence of overweight and obesity in India (Dasgupta *et al.*, 2015). As SSB sale volumes are declining in mature markets of North America and Western Europe (average 15%) (Andreyeva *et al.*, 2022), they are steadily rising in emerging markets of South Asia, Sub-Saharan Africa and East Asia (World Bank, 2020). Though, the current consumption of SSBs (and other unhealthy foods) is lower in South Asia than in other regions, the trends are however, rising (Muhammad *et al.*, 2019).

India is the largest consumer of sugar in the world (Ministry of Consumer Affairs, Food and Public Distribution, 2022) and in 2021 was also the largest producer (27.2 million tons). While the global average consumption of sugar is 22 kg/person/year (OECD and Food and Agriculture Organization, 2021), an average Indian consumes 25 kg/year (sum of regular sugar, free sugar from SSBs and traditional sources such as jaggery). This is five times the WHO (World Health Organization, 2015) recommended threshold for free sugar intake (Kharbanda *et al.*, 2018). The alarming rise in sugar consumption in India could be partly attributed to the rising sales of aerated drinks by 22.5% and rise in all soft drinks by 24.8% from 2016 to 2019 in India (John *et al.*, 2022). Besides sugar, high in fat, sugar or salt (HFSS) food products account from 10% to 30% of the average total caloric intake in rural and urban households, respectively (Sharma *et al.*, 2020). Per capita consumption of sugar in India has risen from 22 g/day in 2000 to 55.3 g/day in 2010, consumption of table salt from 9 g to 12 g/per-capita/day; and per capita fat consumption increased from 21.2 g/day in 2000 to 54 g/day in 2010 (Economic Advisory Council India, 2012). The food processing industry is one of the fastest growing sectors of the Indian economy and accounts for consumption of 50–60% of edible sugar, salt and fats (Dasgupta *et al.*, 2015).

The WHO recommends taxation as one of the most cost-effective tools for addressing population levels of obesity and other related non-communicable diseases (NCDs). Taxation has been used by various countries, in recent decades, to reduce the consumption of SSBs and HFSS (World Bank, 2020), (Jaacks, 2019; Dodd *et al.*, 2020; Pfinder *et al.*, 2020). Over 70 countries have reported SSB taxes at national, regional or sub-regional levels with positive impacts on consumption and reformulation (Backholer *et al.*, 2018; Hattersley *et al.*, 2020). Taxation on SSBs decreased consumption of taxed beverages (and increased purchase of bottled water) in the first year of implementation in Mexico, and continued to the second year (Colchero *et al.*, 2016; 2017) and reduced mean BMI in the younger age groups (Schwendicke and Stolpe, 2017). Another recently published study in *The Lancet Planetary Health* demonstrated that South Africa’s sugar-based Health Promotion Levy (HPL), 10% additional taxes on SSBs (6% increase in prices) resulted in a 16% reduction in volume purchased in first year and to a 51% reduction in use of sugar in beverages, and a 28% reduction in volume of taxed beverages purchased per person per day, as compared to the trends before the tax’s implementation (Stacey *et al.*, 2021).

Global consumer demand modelling studies of SSB intakes and prices (by age, sex and country) have also estimated that a 20% tax (price increase) would result in reductions of SSB intake across countries of varying income level, with particularly significant reductions expected among young adults (Muhammad *et al.*, 2019). Besides reducing consumption, taxes on sugary soft drinks, when the tax rate is tied to the volume of sugar, may encourage manufacturers to reformulate and reduce the amount of sugar in the drinks they produce (Griffith *et al.*, 2021). In India, Basu *et al*. (Basu *et al.*, 2014) noted that if the linear secular trends in SSB consumption continued in the absence of tax, the overweight and obesity prevalence in India was expected to rise from 39% to 49% and type 2 diabetes incidence was expected to rise from 319 to 336 per 100 000 per year over 2014–23. A recent study from India using consumption data from 2011, reported that for a 10% decline in consumption of SSBs in India, the tax rates would need to be increased to 57% [28% goods and service tax (GST) plus 29% cess^2^] from the current 40% (28% GST plus 12% cess) (John *et al.*, 2022).

Though taxation on SSBs is recommended as a cost-effective intervention to prevent and control NCDs (World Health Organization, 2017), the existing GST in India does not differentiate between healthy and unhealthy beverages. It is not based on any public health evidence, but primarily to contribute to the national revenue streams. Previous studies from India used consumption data from 2011 and focused only on SSBs and did not model the impact of health taxes (HTs) on government revenues.

This paper specifically explores how taxation could help reduce the potential consumption of sugar, of SSBs and of foods HFSS. Towards this, our study estimated the price elasticity (PE) for sugar and then proceeded to present various simulations and scenarios (within each simulation) to show the potential impact of increase in taxes on the demand for sugar and sugary foods. We further illustrate the impact of these tax increases on revenue generation for the Indian government.

## Materials and methods

In this study, sugar is defined as all forms of refined and unrefined sugar and gur (brown cane sugar) commonly consumed in households and includes sugar used by bulk manufacturers for all unbranded and unlabelled sweets and confectionaries in India. Sugar sweetened SSBs are any non-alcoholic beverages that contain added sugar or added sweetener such as soft drinks, juices, flavoured milk and milk-based products. HFSS products are processed foods with high levels of total fat or trans-fat or total sugar or salt such as pre-packed branded foods. In this study, we assume that additional HTs are added to the current goods and service taxes resulting in an increase in final prices to the consumers. The resultant increase in prices depends on the proportion of existing tax to the final price. This is slightly different from models which assume a full passthrough of taxes to prices.

Our analysis proceeded in three steps. First, for a hypothetical price of Rs. 100 before taxes, we calculated the end consumer price using the current tax rates (GST for 2022). Then, we estimated the potential impact of adding 10–30% HT to the existing tax rates on the consumer prices of sugar, SSB and HFSS (see Table 1). Finally, we estimated the potential impact of these price increases on the demand for these products.

**Table 1.** Percentage change in prices with proposed tax increase for SSB and foods HFSS

	Hypothetical base price before tax (in Indian Rupees (INR)	Current tax rates for 2022 (%)	Hypothetical consumer price (INR)	Proposed increase in tax (in percent-point)	Proposed tax rates (%)	Estimated consumer price F = A + E (INR)	Percent increase in consumer prices G = (F-C)/C (%)
Product	A	B	C	D	E	F	G
Sugar	100.00	18	118.00	10	28	128.00	8
				20	38	138.00	17
				30	48	148.00	25
SSB^a^	100.00	28	140.00	10	50	150.00	7
		12		20	60	160.00	14
				30	70	170.00	21
HFSS	100.00	12	112.00	10	22	122.00	9
				20	32	132.00	18
				30	42	142.00	27

a A 12% cess in addition to GST of 28%.

### Data sources for PEs

We estimated the PE for sugar using Private Final Consumption Expenditure (PFCE) and Consumer Price Index (CPI) data. PFCE is macro-level data compiled annually as part of the National Accounts Statistics and represents consumption expenditure of all households of the country irrespective of their characteristics. The CPI is also macro data that represents retail prices for the consumption items in the country. Both data are published by Ministry of Statistics and Programme Implementation of Government of India. Sugar and gur (brown cane sugar) are clubbed together in PFCE and CPI, and we have used this category for the model. A time-series data for 26 years (1984–85 to 2011–12) at constant prices was used for modelling purposes. The analysis was conducted up to 2011–12 as the PFCE data beyond this period included other products like confectionaries, honey, etc. in the same category apart from sugar and gur. Due to data limitations, we used simple econometric modelling. A log–log ordinary least square regression was used to obtain price elasticity through a partial equilibrium model. This elasticity estimate reflects how the demand for sugar changes with price movement.

The simple regression equation was transformed into a double log function of the type:


(1) $$In\left( {consumption} \right){\ } = {\ }In{\ }a{\ } + {\ }{b_1}*ln\left( {price} \right)$$

In this case, the regression coefficient *b*_1_, is the own PE that measures changes in the dependent variable (consumption expenditure on sugar and gur) due to change in the independent variable (price of sugar and gur). The model goes through several specification tests to determine robustness and significance of the model. Once the elasticities are estimated through modelling, changes in the dependent variable can be predicted.

For SSB and HFSS, we used own-price elasticities obtained from an in-depth review of published literature from Indian and International studies. We considered the lowest and highest own price elasticities to establish a range for our analysis. This in turn helped us arrive at a range of reductions in demand due to an increase in taxes.

- For sugar, we used the estimated elasticity (through the process described earlier). To project the potential impact of the increase in price of sugar on manufacturers of confectionery goods, we opted for a maximum of 4-fold increase in PE for sugar as part of the simulation model. This is assuming that the confectionery manufacturers who purchase up to 55% of the annual sugar produced in India (Ministry of Consumer Affairs, Food and Public Distribution, 2022), may be more sensitive to prices of sugar than a household that purchases smaller quantities as part of their essential food basket.
- For SSBs, we considered three levels of PEs: (i) −0.94, representing average Indian context (Basu *et al.*, 2014; John *et al.*, 2022), (ii) −0.60, representing minimum elasticity from international studies, Saudi Arabia (Alsukait *et al.*, 2020), (iii) −1.39, representing maximum elasticity from international studies, Guatemala (Piñeiro *et al.*, 2019).
- For HFSS, we considered two levels of price elasticities as there was no reference found in the Indian context and minimal international references. (i) −0.53 as in Mexico (Batis *et al.*, 2016) and −0.87 as in Hungary (Bíró, 2015).

For all three types of food products (sugar, SSB and HFSS), we then estimated changes in levels of demand by multiplying the rate of price change (resulting from increase in taxes) with price elasticity estimates for the respective products.

In addition, we estimated the impact of varying tax rates on revenue using the estimated impact on demand adjusted for the increase in taxes:


(2) $$\begin{aligned} &Impact{\ }on{\ }revenue{\ }\left( i \right){\ } = {\ }\left( \left( \left( {1 - change{\ }in{\ }demand} \right) \right.\right.\nonumber\\ & \quad \left.\left.X{\ }proposed{\ }tax{\ }rate \right){\!}-{\!}original{\ }tax{\ }rate \right)/original{\ }tax{\ }rate \end{aligned}$$

This enabled identification of tax rates that would enable decrease in demand for foods high in, fats, salt, or sugar in India, while increasing the total revenue generated from taxes of these products.

## Results

The study used a simple double log-regression model and estimated the own PE of sugar to be—0.20 [95% confidence interval (CI) −0.15 to −0.34] (Table 2). The model explained ∼53% of the data variations. The overall own PE of −0.20 for sugar implies that if the price of sugar is increased by 10%, demand for sugar will be reduced by 2% with all other factors driving the demand remaining constant.

**Table 2.** Sugar price elasticity using regression model^a^

Variables	Coefficient	Standard Error	*t*	*P*> | *t*|	95 Confidence interval
Price of sugar	**−0.20**	0.046	5.21	0.0000	0 0.15–0.34
Constant	9.31	0.23	41.4	0.0000	8.84–0.77

a Estimated using Private Final Consumption Expenditure and Consumer Price Index 1984–85 to 2011–2012.

Table 3 provides estimates of changes in demand for sugar, SSBs and HFSS along with the potential changes in tax revenue that are expected as a result of the increase in tax rates and therefore prices of these products. Within each simulation (in Table 3), three separate scenarios are presented for varying levels of PEs, illustrating three policy options for the government in terms of three different proposed tax rates. The proposed taxes may be levied as a HT to existing GST tax rates for these products in India.

**Table 3.** Impact of taxes and price elasticity on the demand for SSBs) and foods HFSS

Products	Proposed tax rates (10–30% additional health tax) (%)	Estimated increase in consumer price (%)	Expected change in consumer demand			Expected change in revenue		
**Sugar (current tax 18%) (%)**			**PE for sugar^a^**					
			**−0.20**	**−0.50**	**−0.70**	**−0.20**	**−0.50**	**−0.70**
	28	8	−1.7	−4.2	−5.9	52.9	49.0	46.3
	38	17	−3.4	−8.5	−11.9	104.0	93.2	86.1
	48	25	−5.1	−12.7	−17.8	153.1	132.8	119.2
**SSB (current tax 40%) (%)**			**PE for SSB^b^**					
			**−0.60**	**−0.94**	**−1.39**	**−0.60**	**−0.94**	**−1.39**
	50	7	−4.3	−6.7	−9.9	19.6	16.6	12.6
	60	14	−8.6	−13.4	−19.9	37.1	29.9	20.2
	70	21	−12.9	−20.1	−29.8	52.5	39.8	22.9
**HFSS (current tax 12%) (%)**			**PE for HFSS^b^**					
			**−0.53**	**−0.87**		**−0.53**	**−0.87**	
	22	9	−4.8	−7.8		74.6	69.0	
	32	18	−9.5	−15.7		141.2	124.9	
	42	27	−14.3	−23.5		199.9	167.8	

a PE for sugar was estimated to be −0.20, we assumed −0.50 and −0.70 to show potential PE for sugar for manufacturers of confectionaries.

b PEs for SSB (−0.60, −0.94, −1.39) and HFSS (−0.53 and −0.87) are from the literature.

Change in demand= PE × percent change in prices.

Change in revenue = (((1−change in demand)* new tax rate)—original tax rate)/original tax rate.

### Tax impact for sugar

We estimated that at a price elasticity of −0.20, an additional 10% HT to the existing GST of 18% (total tax 28%, 18% GST plus 10% HT) on sugar will result in a price increase of 9%, which translates to a 2% decrease in demand for sugar (−0.20*9% = −1.8%). Similarly, a 30% additional HT (48% total tax) on sugar will result in a price increase of 25%, and with PE of −0.20, this will translate to a 5% decrease in demand of sugar (−0.20*25% = −5%). At higher PEs of −0.50 and −0.70 (possibly for bulk consumers of sugar), additional HTs of 10–30% would reduce the demand for sugar by 4–13% and 6–18%, respectively.

### Tax impact on SSBs

With the price elasticities for SSBs ranging from −0.60 to −1.39, an additional HT of 10% resulting in a total tax of 50% (28% GST + 12% cess + 10% HT) would result in a 7% increase in prices for SSBs resulting in 4–10% reduction in demand of SSBs. Similarly, an additional health tax of 20% (28% GST + 12% cess + 20% HT) would result in a 21% increase in prices for SSBs leading to a 13–30% reduction in demand of SSBs.

With a PE of −0.94 in the Indian context, 10% additional HT on SSB would result in a 7% decline in SSB demand (−0.94*7% price increase =−6.7%). Similarly, an additional HT of 30% (total tax 70%: 28% GST + 12% cess + 30% HT), would result in a 21% increase in prices for SSBs, resulting in a substantial decline of over 20% in demand (Table 2). Considering the lowest PE of −0.60 (in Saudi Arabia), proposed total tax rates of 50–70%, can reduce demand by 4–13%. If we consider a much larger PE of −1.39 (as seen in Guatemala), the proposed taxes of 50–70%, could result in a 10–30% decline in the demand for SSBs (Table 2).

### Tax impact on foods HFSS

For PEs of HFSS ranging from −0.53 (in Mexico) to −0.87 (in Hungary), an additional 10% health tax (total tax 22%, 12% GST + 10% HT) would result in 9% increase in prices resulting in a 5–8% reduction in demand of HFSS products. Similarly, an additional HT of 30% (total tax 42%, 12% GST + 30% HT) would result in 27% increase in prices resulting in a 14–24% reduction in demand for HFSS food products.

### Impact on tax revenues

The proposed taxes on sugar, SSBs and HFSS are estimated to result in an increase in tax revenues ranging from 13% to 200% across various scenarios (Table 3). For SSBs, an additional HT of 10–30% for the three different PEs (−0.60, −0.94, −1.39), the tax revenue is expected to increase between 13% and 53%. For sugar, the increase in revenue ranges from 46% (PE—0.70, tax 28%) to 153% (PE—0.20, tax 48%), while increasing taxation of HFSS from 12–22% to 42% will result in increase of tax revenues from 69% to 200%.

## Discussion

Our study demonstrates the potential impact of additional HTs on the demand for sugar, SSBs and HFSS in India. Sugar, an essential commodity for an average Indian household, as expected has a low-PE (−0.20). A 10% additional HT (total tax 28%, 18% GST plus 10% HT), would increase the retail price by 8% which may result in a small decline (2% in the annual demand for sugar). Government may either choose this as a policy option for households so the price increase is minimal or may choose not to change tax rates for sugar purchased by households. Given that manufacturers of confectionery products are the largest consumers of sugar and may be more sensitive to price changes, as these would impact their input cost and profits, we propose, as a policy option, the use of higher HT rates of 20% or preferably 30% for bulk consumers, like the manufacturers of sweets and confectionaries. Our model shows that a 20–30% additional HT (total tax of 38% or 48%) for bulk consumers of sugar (manufacturers of confectioneries and sweets) (assuming −0.70 PE), would result in 17–25% price increase, thereby resulting in 12–18% potential decrease in the demand for sugar. Any such increase in tax rates, we assume, would be mostly passed on to consumers which then would have a significant impact on the demand of sugar-based confectioneries in India.

**Figure 1.**
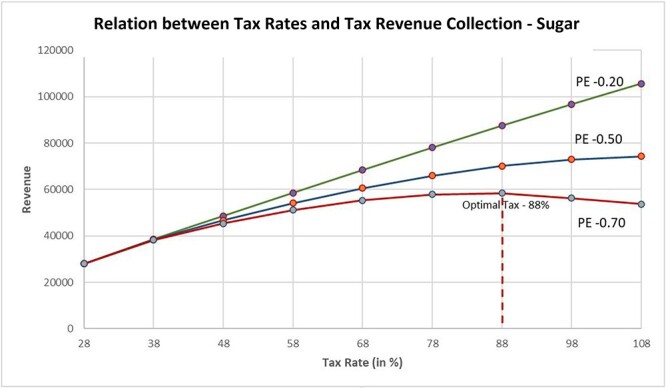
Impact on tax revenue with increase in taxes for sugar with increasing prices (5% annually) and demand (2% annually)

Our calculation of tax revenue shows that this decline in potential consumption, however, will have no negative effect on tax revenues, rather tax revenues could potentially increase by 46–153%. This is primarily because of the nature of these products which have PE less than one due to which although prices increase as a result of additional taxes, the reduction in demand is not high enough to decrease the total revenues. In addition, since the price increase is a result of additional taxes, the tax component of the revenue increases and the combined effect is an increase in the tax revenue obtained by the government. These calculations assume that all other factors like income levels and base manufacturer price remain the same. We estimated the impact by considering the possibility of these variables changing marginally over the years and Figure 1 shows that in most scenarios, there is no negative impact on tax revenues. For PE rates of −0.70, assumed for manufacturers buying sugar in bulk, a small decline in revenue seems possible when tax rates reach around 88% (Figure 1). Thus, it seems that at the proposed additional HT of 10–30%, while there would be a decline in demand for sugar especially for manufacturers of sweets and pastries, there would be a significant increase in tax revenues for the government. These additional revenues could be used for cross subsidizing sugar cane farmers, or providing subsidy/incentives for both producing and consuming fresh fruits, and/or improving health programmes, etc.

For SSBs, our model shows that an additional health tax of 10–30% (total tax rates of 50–70%) would result in a 7–20% decline in the demand of these products (at PE of −0.94). This is similar to findings reported by John *et al*. who estimated that a tax rate of 57% (28% GST plus 29% HT) is required or a 10% decline in the demand for SSBs. Recent study by Muhammad *et al.* reported that for global PEs for SSBs (ranging −0.4 to −1.25), a potential impact of 20% tax, assuming 20% increase in prices (Muhammad *et al.*, 2019) could decrease demand for SSB from 15% to 25%. These findings resonate with our analysis of similar PE ranges and where a 21% price increase is estimated to result in 10–30% reduction in demand. However, this would have no negative impact on tax revenues, rather, we estimate that tax revenues would keep rising even at 80% tax rates for these products.

For HFSS products, our analysis showed that at 30% additional HT rate (total tax 42%, 12% GST + 30% HT) with a PE of −0.87%, would result in 24% decline in demand for HFSS products. This decline in demand, however, does not result in any decline in tax revenues, rather our analysis shows that even with an increase in prices (5% annually) and demand (2% annually) tax rates can be increased up to 52% (40% additional HT) with no negative impact on tax revenues.

In addition to the modelled impact, there are now several studies that clearly show evidence of an actual decline in demand for SSBs with the introduction of additional health taxes. In Chile, with tax rate increase of 18% in SSBs, the household monthly per capita consumption decreased by 3.4%%, 1-year post-tax implementation (Caro *et al.*, 2018). In Mexico, with a PE of −1.16, by imposing 1 peso per litre of excise tax on all SSBs, a reduction of 6.3% in SSB purchases was observed 2 years post-implementation of taxes (Colchero *et al.*, 2017). In Saudi Arabia, a 50% SSB tax resulted in a 19% decrease in consumption of SSBs within a year (Jalloun and Qurban, 2022).

The impact of taxation on health can be maximized by combining with interventions and a range of complementary strategies that improve population diets. Taxes on foods high in fat, salt, or sugar is one effective policy lever and can be implemented alongside other evidence-based policies, such as marketing restrictions on unhealthy food and beverages, interpretive front of pack labelling schemes, policies for healthier school food environments, increased access to healthy food and beverages like clean water (unbottled), fresh fruit, fruit juices (without added sugar), etc. To minimize the chances of substitution of sugar with non-nutritive sugar supplements and artificial sweeteners, we recommend that all alternatives of sugar be taxed at the same rates as sugar. In the long term, HTs when implemented in a comprehensive suite of obesity prevention policies would translate to a decrease in obesity, related NCDs in India.

In addition, as an international best practice, to address increased affordability risks due to per capita income growth (for example, GDP per capita growth) and inflation, it is recommended to apply taxes that are regularly adjusted for increases in the retail prices as a result of inflation and average household incomes (World Bank, 2020). A national-wide campaign on healthier eating and extensive research on calculation of portion size and amounts of sugars consumed is also highly recommended (Gulati and Misra, 2014).

Our study used available data on PEs; however, we noted the sparsity of data on price elasticity for sugar, SSBs and for HFSS products in the Indian context. We were unable to find reliable PE estimates for sugar and estimated it using best available data in India, from 2011 to 2012. The paper used a log–log regression model with available PFCE data and the CPI data to estimate the PE of sugar. This estimate may not be the most accurate; however, given the essential nature of sugar for households, our estimate of −0.20 seems reasonable. For PE of SSBs and HFSS, the studies depended on PEs as calculated by published studies from India and across the globe, and are not from very recent studies. The price elasticities may have changed over time and would be different for different income levels and for different age groups. We have not modelled these variations; however, we believe the policy impact of tax increases is not overestimated or overstated. Our study estimates show that the additional HTs of 10–30% are expected to increase consumer prices by 7–27%. This estimated impact of additional taxes on consumer prices and then on demand may be slightly less than other studies that assume a full pass-through effect of taxes on prices. However, in reality, a full pass-through effect is rarely achieved as the tax component of a product is rarely <5%.

In spite of recognizing the rising epidemic of obesity and NCDs in India, current government policies benefit the processed food industry directly. These include supply-side factors such as water availability, low-cost labour, product manufacturing and packaging technology enhancements and so on (Dasgupta *et al.*, 2015). Adolescents and young adults are the highest consumers of SSBs and HFSS foods (P. Gupta *et al.*, 2019; Pries *et al.*, 2019) and the long-term health impacts of the consumption of SSBs and HFSS on our young generation should be a deciding factor for our policy makers. Basu *et al*. estimated that in India, 20% increase in prices of SSBs may reduce overweight and obesity by 3% and type 2 diabetes by 1.6% with largest relative effect expected among young rural men (Basu *et al.*, 2014). A recent study that modelled the impact of sugar intake on dental caries showed that a 20% increase in price of sugar could prevent 27.96 million tooth-loss incidents among the population cohort of India (A. Gupta *et al.*, 2022).

We thus recommend that an additional **HT of 20–30%** (adjusted annually to inflation and increase in per capita income) **in addition to GST may be considered for sugar, SSBs and HFSS foods in India:** 20% additional HT (total tax 48%, 18% GST plus 20% HT) for bulk consumers of sugar, like the manufactures of sweets and confectionaries; 20% HT (total tax 60%, 28% GST +12% cess+ 20% HT) for sugar sweetened beverages, and 30% HT (total tax 42%, 12% GST plus 30% HT) for foods HFSS. This, when combined with a nationwide campaign for healthier eating options and lifestyle change (funded by the increased tax revenues), would result in important long-term benefits to the health and wellbeing of the people in India.

## Data Availability

We used a data set for estimating price elasticity of sugar, it is publicly available. We have provided the details in the text. Rest are from the literature and our model is sufficiently clear for anyone to replicate.

## Footnotes

1. Within the definition of costs, ‘direct’ and ‘indirect’ costs (including societal costs) are applied.

2. Cess is a form of tax over and above the base tax liability of a taxpayer. It is usually imposed additionally when the government intends to raise funds for specific purposes like education, health, and the similar ones.

## Reflexivity statement

The authors include six women and three men with professional experience spanning across multiple levels. While all authors specialize in various areas of public health and health policy, specialize in Nutrition, four in health systems, one is a statistician, and another one is closely involved with policy development in the public sector. This study has benefitted greatly from the deep expertise of the authors in India, and their work across varied geographies within public health practice, economic modelling and analysis, research and policy making.
